# From centralized DRG costing to decentralized TDABC-assessing the feasibility of hospital cost accounting for decision-making in Denmark

**DOI:** 10.1186/s12913-021-06807-4

**Published:** 2021-08-18

**Authors:** Margit Malmmose, Jogvan Pauli Lydersen

**Affiliations:** 1 Department of Management, Aarhus School of Business and Social Sciences, Aarhus University, Fuglesangsalle 4, 8210 Aarhus V, Denmark; 2 Cand.Oecon, Ringkjøbing Landbobank, Torvet 1, 6950 Ringkøbing, Denmark

**Keywords:** Hospitals, Cost accounting, DRG, Value-based healthcare

## Abstract

**Background:**

The objective is to examine hospital cost accounts to understand the foundation upon which healthcare decisions are based. More specifically, the aim is to add insights to accounting practices and their applicability towards a newly establish value-based agenda with a focus on patient-level cost data.

**Methods:**

We apply a cost accounting framework developed to position and understand hospital cost practices in relation to government requirements. Allocated cost account data from 2015 from all Danish hospitals were collected and analyzed. These cost accounts lay the foundation for diagnosis related group (DRG) rate setting. We further compare the data’s limitations and potential in a value-based healthcare (VBHC) agenda with the aim of implementing time-driven activity based costing (TDABC).

**Results:**

We find exceedingly aggregated department-level data that are not tied to patient information. We investigate these data and find large data skewness in the current system, mainly due to structural variances within hospitals. We further demonstrate the current costs data’s lack of suitability for VBHC but with suggestions of how cost data can become applicable for such an approach, which will increase cost data transparency and, thus, provide a better foundation for both local and national decision-making.

**Conclusions:**

The findings raise concerns about the cost accounts’ ability to provide valid information in healthcare decision-making due to a lack of transparency and obvious variances that distort budgets and production-value estimates. The standardization of costs stemming from hospitals with large organizational differences has significant implications on the fairness of resource allocation and decision-making at large. Thus, for hospitals to become more cost efficient, a substantially more detailed clinically bottom-led cost account system is essential to provide better information for prioritization in health.

**Supplementary Information:**

The online version contains supplementary material available at 10.1186/s12913-021-06807-4.

## Background

According to accounting theory, managers must know the costs of an organization’s resources and activities to make correct and timely decisions [1–3]. The importance of cost accounting for decision-making has also been raised in hospital settings [4, 5], where in later years, time-driven activity-based costing (TDABC) as an accounting method has been highlighted to support such notions [6–8]. Lacking emphasis on cost accounting in a hospital setting ultimately leads to someone not receiving the care they need. However, research shows that cost information is seldom applied in practice in hospitals and that cost methods vary dramatically [9, 10].

This paper addresses cost accounting in Danish hospitals with the aim of specifying its feasibility for decision-making. Cost accounting measures, analyzes, and reports financial information relating to acquiring and using resources. Studies show that costing data must be adequate and in a standardized format to support hospital management [5, 11–13]. Research emphasizes the hospitals’ cost accounting systems as a pertinent managerial foundation because it informs hospitals’ and central authorities’ decisions and, thus, has a significant influence on decisions made in hospital organizations, therefore influencing treatment choices and availability [6, 7, 12, 14–16]. Additionally, continuous increasing health expenditures [17, 18] sustain pressure on hospitals for cost containment [5, 12–14, 19], further supporting a concern with cost accounting.

Theoretically, cost accounting has long been part of the foundation for diagnosis-related group (DRG) rates, which are applied in most OECD nations for benchmarking, pricing, and/or hospital resource allocation [10, 12, 20]. Moreover, cost accounting is a vital part of the newly defined value-based healthcare (VBHC) agenda [21, 22], which seeks to investigate the patient’s total use of resources across healthcare departments and organizations [9, 23–26] through the particular application of TDABC [6, 16, 27, 28]. Additionally, cost accounting is highlighted to be of vital socio-political importance for “solving the cost crisis in healthcare” [29] and achieving cost-effective delivery of healthcare [5, 14]. Thus, cost accounting is positioned to be an important, although often neglected [13], managerial as well as political steering, control, and evaluation tool.

This importance is supported by Chapman et al. [13] and Kaplan et al. [9], who emphasize the significance of evaluating costing practices and their relation to the wider context. Thus, we develop a new examination on previous studies of Raulinajtys-Grzybek [30] and Ankjær-Jensen et al. [31], who investigated the Danish cost accounting model in 2005–2006 that established the foundation for DRG rates and production-value calculations applied for resource allocation. Both studies found challenges with the cost accounting, which was highlighted as lacking transparency. However they didn’t show the direct calculative challenges. Additionally, Tiemann [32] finds that the Danish hospital data are highly aggregated and, therefore, had to be excluded from the cross-country analysis of hospitalization costs within acute myocardial infarction and was also indicated by a large number of overhead costs compared to other nations [5]. Additionally, the National Audit Office of Denmark highlighted concerns with the alignment and transparency of the costing information from hospitals [33]. The cost accounting comprised 3.14% of Danish GDP in 2015 [34]. Thus, the methods for allocating these costs and their influence on sector-level decision-making is crucial in relation to healthcare cost-effectiveness and decision-making. Assessing new data for the entire nation, we will take these previous analyses and conclusions further, where we aim at illustrating how and to what extent the current data are useful and specifically illustrate the data and accounting approach necessary to improve the quality of the current cost account data.

Not surprisingly, the findings illuminate discrepancies in the current cost account procedures. Most obvious is the aggregate level of data that do not appear to be associated with patient data prior to or during the cost allocations, which is problematic in establishing patient-level cost data [9]. Thus, there is a central versus decentralized challenge, which plays an important role in understanding data and its feasibility correctly. For example, our findings clearly show local differences in cost allocation as well as structural set-ups, which skew the data to be centrally comprised and standardized. Although some of the current set-up may be useful for understanding cost application on a broad national level, it is not feasible for local controllability and decision-making. We show how the application of TDABC locally emphasizes employee and equipment activity assessment, which will provide a substantially more transparent approach to local decision-making.

### The costing side of VBHC

There is a growing body of literature investigating the phenomenon of value-based healthcare initiatives in hospitals [23, 25, 26, 35, 36], which particularly focuses on the more strategic elements of providing services from a patient perspective [35–37]. Another stream of literature focuses on the implementation of TDABC in relation to VBHC [7, 16, 27, 28, 38], where most of these studies have to narrow the scope of study down to department or facility level to comprehend the complexity [38, 39]. The TDABC approach is a simplified and extended strategy of the traditional activity-based costing (ABC) approach introduced as a costing method in hospitals during the 1990s and 2000s [40–43]. A major difference between the ABC/TDABC approach and DRG rates is the decentralized use of ABC/TDABC and the central application of DRG rates as a prospective accounting tool used for national pricing, budgeting, and benchmarking [20, 44]. We acknowledge these differences in the pursuit of investigating a current national cost account system, which healthcare top managers and governance bodies wish to change to a value-based approach system [45, 46]. In this respect, we investigate the transparency and feasibility of current cost data available in hospitals in Denmark with the aim of applying these data for further developing a patient-level cost data approach [46] in the current transiting from volume- to value-driven healthcare [47].

Thus, our objective is to examine the available hospital cost accounting information to understand hospital- and sector-level implications as well as the feasibility for future value-based healthcare application. We specifically evaluate the current set-up, allocation methods, applied locally by the hospitals, which is then distributed to sector-level application for benchmarking and resource allocation. We then compare this assessment with the TDABC approach, which is recommended as part of the value-based agenda [4, 22]. This comparison is achieved through an illustration of how the data could be set-up and what type of information this set-up demands. Finally, we discuss the concomitant implications of the current system as well as the transit towards a more refined cost accounting system. We loosely apply the concepts of Tan et al. [12] to understand the level and detail of cost data. Further, we apply the accounting technical approach of TDABC [6, 27] to illustrate the current cost accounting’s feasibility.

### The case of Denmark

In Denmark, DRG rates have been in place since 1996 [48]. The application of DRG rates in controlling and evaluating the hospitals has been continuously developed. Since 2004, DRG rates have been applied to calculate yearly productivity reports.^1^ With the introduction of a structural reform, which restructured the Danish healthcare landscape in 2007, the focus on DRG rates and productivity became further sustained [49–51]. All hospitals became part of a geographical region, which is administratively in charge of running the Danish hospitals [52]. A baseline was implemented, which is a value-estimated budget reflecting the activity that the hospitals are required to perform. The baseline is a goal for the activity that the hospital should perform within a given year. The regions calculate this baseline by multiplying the hospital’s output with the current DRG rates for the specific outcomes. Thereby, the activity that the hospitals have to perform can be expressed in financial values [33]. Cost accounts provide one part of the foundation for DRG rate calculations which is matched with patient activity from a different database [53]. The dataflow that has provided the DRG inputs for over a decade in Denmark is illustrated below:

The Danish National Patient Registry, as illustrated in the figure, has drawn tremendous research attention and potential due to its detailed registration of patient activity [33, 54]. The cost accounting is, in contrast to the patient activity data, completed by back-office management accountants, who are not in contact with the patient administrative systems; hence, the preparation of the cost allocation is decoupled from the direct activity registration at the hospital level and, accordingly, has drawn little attention, as has the matching of cost data with patient data in the national cost database.

Within the last 5 years, there has been an increasing wish and attempt to disregard productivity and DRG rates as steering elements and, instead, pursue the international trend of value-based management [47, 55, 56]. Different local attempts of implementing non-financial performance measures to support a patient focus have been initiated [57–59]. In a similar vein, there has been a change in the managerial accounting focus, where in 2017, a wish towards more reliable and valid accounting data has been stated and an analysis initiated to map such a need and its application [45]. In this assessment done by the Health Innovation Institute, it is highlighted that the Danish hospitals are generally good in assessing budget data, which were found to be accessible and valid. However, they particularly stress issues with resource planning and capacity control. Finally, they suggest that data solutions should be developed in close co-operation with clinicians. Additionally, the Health Data Authority asked McKinsey to evaluate and investigate the potential of the current cost database as a foundation for value-based healthcare management [46]. They conclude that there is potential for the cost database to be applied for value-based management because it applies a ‘bottom-up’ approach, and the quality of the data is good on an aggregate level (p. 1). They particularly highlight the lack of patient cost data. However, the McKinsey assessment is rather superficial. It does not illustrate the challenges, nor does the report properly explain what initiatives could be taken to overcome those challenges. Finally, there is no understanding or reference to TDABC, which is a vital accounting technique for supporting value-based management [6, 16, 27]. Thus, at this point, little is known about the concrete accounting data challenges as well as how to overcome these challenges, which warrant an assessment of the cost account data.

## Methods

**Fig. 1:**
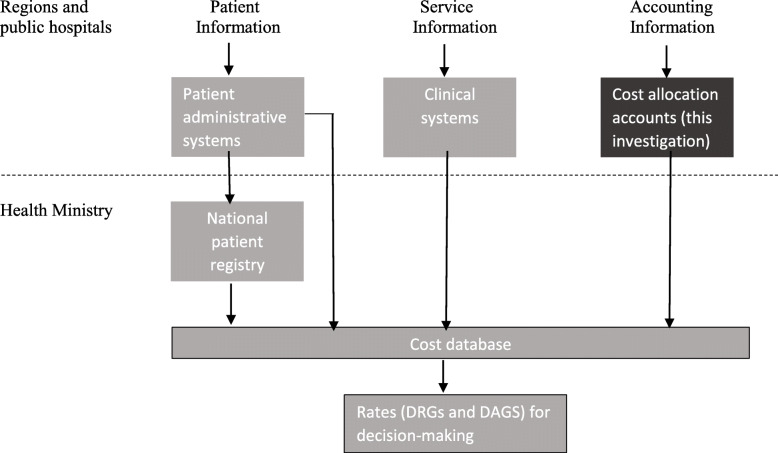
Dataflow for DRG and DAGS calculations. Regions and. Patient. Service. Accounting public hospitals. Information. Information. Information. Source: The National Audit Office of Denmark [14]

We take a technical accounting approach and acknowledge accounting as a socio-technical discipline that centers specific techniques and logics constantly developed, shaped, and influenced by the social context [60]. Therefore, we depart from the current cost accounting system in Denmark (as the context) which is first overall assessed and depicted. In doing so we examine whether the cost data is detailed enough to match patient data from the National Patient Registry (see Fig. 1). Second, to illustrate details and understand its fit and/or differences from TDABC, we extract one type of surgical procedure—eye surgeries. We illustrate the cost accounts from two different hospitals to illustrate their differences and, therefore, the method’s challenges in the current system. From that point, we explicate the possible application of TDABC to one of these hospital’s eye surgical procedures and the hospital’s departments in general, and then we provide an overview of the differences in the current approach and TDABC. Finally, we discuss future challenges in pursuing the aim of value-based healthcare with the present available data. Thus, the results are presented in 3 steps: 1) an overall assessment of the current cost accounting, 2) comparison of two specific departments in the current cost accounting system, and 3) taking one of the departments to illustrate how TDABC could be applied to enhance cost transparency and, thus, controllability supporting better decisions.

Tan et al. [12] describe four approaches for calculating the direct department costs per patient. These approaches are placed in a matrix *identifying* hospital service accuracy on one axis and accuracy of *valuating* the services on the other. Micro-costing *identifies* the accuracy of hospital service with high valuation accuracy, whereas gross-costing is less accurate in identification than micro-costing. On the other axis, we have top-down versus bottom-up, where top-down values inpatient days per average patient (less identification of hospital service), and bottom-up values inpatient days per actual individual patient (better identification of hospital service). We apply these concepts to distinguish between the current approach and TDABC.

### TDABC

According to Kaplan [6], “Sustainable cost reductions must start with clinician-led, bottom-up re-engineering that enables providers to maintain and improve their healthcare outcomes while reducing the costs of delivering that care” p. 82. He suggests a simple model for this approach: Resource cost (C) = Quantity of resource units (Q) x Price per unit of resource (P). This is a simple formula that can be applied to all resources acquired, whereby almost all personnel, equipment, facilities, and indirect and support costs can be directly attributed rather than indirectly allocated. The steps are specified to be [6, 28]:

TDABC steps:

1. Select the medical condition
2. Define the care delivery value chain – chart the key activities
3. Develop a process map that includes each activity and incorporate all direct and in-direct resources
4. Obtain time estimates for each process (activities and resources used)
5. Estimate the cost of each resource
6. Estimate the capacity of each resource and calculate the capacity cost rate
7. Calculate the total cost of patient care

TDABC as part of VBHC examines the entire patient care path’. However, in this study, we narrow the focus to hospital costing data and, thus, for analytical purposes, exclude patient costing data from municipalities and other caretakers.

### Data

This study evaluates all Danish hospitals’ cost allocation accounts from 2015, which are distributed to the Danish Health Data Authority by the hospitals. These are the cost accounts illustrated on the local level to the right in Fig. 1. These data are at a later point combined with detailed patient data (shown in Fig. 1 to the left) from where DRG rates are calculated. All Danish regions were contacted in 2017, and during the following months, the authors received all hospitals’ cost accounts in Denmark. There are altogether cost allocation accounts from **20 hospital organizations**, some of which comprise several hospitals. The collected cost allocation accounts comprise a master document developed according to a specific guideline centrally developed by the Health Data Authority. Additionally, **three meetings** were held with a hospital cost allocation account responsible in order to clarify the procedures and concepts applied. The guideline report from the Danish Health Data Authority has, moreover, created the foundation for understanding the cost accounting. Finally, some of the issues raised during the evaluation were verified or clarified by a previous consultant at the Danish Health Data Authority.

### The cost account spreadsheet

The cost accounting is generated in a Microsoft Excel document, which comprises eight interrelated sheets: a ***cost map***, where the different cost centers are identified and coded to be either external, support, or a department. The support centers are overhead costs, divided into four different levels predefined by the Health Data Authority as a guided sequential step-down method. A sheet for ***cost allocation accounts*** comprises accounts from the fiscal reporting. Sheet 4 is a guided ***cost driver*** sheet, where the organization can mark the type of cost drivers applied. Sheet 3 comprises the ***support centers*** in the rows, whereas all the cost centers are identified in the columns. It is in this sheet that the actual percentage is inserted, identifying how much of the support center costs is allocated to each final function or department. Here, it is possible to specifically identify which cost driver is applied for each support center allocation; therefore, it is those sheets that are analyzed and evaluated. The remaining four sheets are summaries or guidelines.

The cost accounting Excel sheets include both direct and indirect costs. Direct items are allocated directly to a clinical department, and indirect items are firstly allocated to a support department (e.g., HR) and then to the clinical department. Indirect costs are typically administrative and facility management, which cannot be directly linked to a specific department. Finally, we have the category of hospital overhead costs, which are allocated to the clinical departments.

## Results

### General assessment of all hospital cost accounts

Table 1 shows the aggregated findings of evaluating the 20 hospitals’ cost accounting reports. It can be observed that one region—E—is reporting its hospital costs collectively. The other regions report their hospitals’ cost accounting in four to six reports. Each of these reports comprises, on average, two physical hospital units. The inpatient and outpatient activities were identified as a size and activity indicator. These numbers stem from the actual patient administrative systems, which are used as an activity basis for DRG rate calculations (see Fig. 1). The cost allocation reports show the direct and indirect costs in absolute numbers. The direct costs comprise department costs. Thus, there is a distinction between direct department vs. direct diagnosis costs. In the cost allocation accounts, we find no detailed diagnosis costs. Therefore, the direct costs exist on an aggregated department level, illustrating a gross- rather than micro-costing approach according to Tan and colleagues’ [12] conceptual framework. We furthermore identify substantial variations in activity level and costs. The largest organizational account unit has total costs of nearly 8 billion DKK, whereas the smallest unit only has costs of 438 million DKK. On average, the indirect costs account for 25% of total costs, but on an organizational level, this varies from 21 to 29%. The number of department cost centers varies significantly (from 53 departments/functions to 291 departments/functions). A cost center is a department or function to which costs are allocated [2]. In this case, it is the final cost centers that are illustrated (i.e., clinical departments). These results illustrate large structural differences with variations in the services provided and how the hospitals are organized.

**Table 1:** Overview of cost account data

Organisational unit for cost accounts	Region	In patient activity*	Out patient activity*	Direct costs	Indirect costs	Total ratebased costs	Number of clinical department/function cost centers	Level 1 support cost centers	Level 2 support cost centers	Level 3 support cost centers	Level 4 support cost centers	Total support cost centers	Method applied in allocation of support costs
1	A	115644	819123	5,176,250,045	1,763,911,170	6,940,161,215	216	1	7	20	15	43	Direct method mixed with stepdown method
2	A	83285	348775	2,179,057,665	762,969,006	2,942,026,671	87	1	3	15	1	20	Direct method mixed with stepdown method
3	A	64404	367688	1,890,066,586	740,352,723	2,630,419,309	72	1	3	11	1	16	Stepdown method
4	A	8822	45031	316,528,965	121,973,705	438,502,670	46	1	1	6	-	8	Stepdown method
5	A	115646	770421	3,655,509,804	1,110,991,198	4,766,501,002	111	1	1	18	2	22	Stepdown method
6	A	81151	296014	1,969,489,090	585,319,321	2,554,808,411	100	1	2	2	2	7	Stepdown method
7	B	238620	929026	862,207,044	317,394,975	1,179,602,019	56	1	2	4	-	7	Stepdown method
8	B			2,635,142,417	679,531,497	3,314,673,914	112	2	2	3	-	7	Stepdown method
9	B			2,033,639,571	602,379,841	2,636,019,413	71	1	2	6	9	18	Stepdown method
10	B			632,358,533	256,457,460	888,815,993	66	1	2	1	2	6	Stepdown method
11	C	107418	876507	5,339,372,658	1,496,903,856	6,836,276,514	125	1	3	5	4	13	Stepdown method
12	C	47601	282060	1,382,763,888	449,469,928	1,832,233,815	98	1	3	6	1	11	Direct method mixed with stepdown method
13	C	40655	293221	1,389,776,166	430,673,637	1,820,449,803	148	1	6	5	3	15	Stepdown method
14	C	55374	546770	2,582,136,383	725,974,575	3,308,110,958	120	1	11	15	5	32	Stepdown method
15	D	34454	151967	777,340,559	250,465,965	1,027,806,524	70	1	4	4	3	12	Stepdown method
16	D	52145	145257	1,849,812,191	519,074,864	2,368,887,055	53	1	3	3	1	8	Direct method only
17	D	100258	721965	5,239,508,199	1,419,808,520	6,659,316,720	123	2	8	11	2	23	Direct method mixed with stepdown method
18	D	49959	311920	1,969,438,664	574,918,325	2,544,356,989	147	1	6	7	1	15	Stepdown method
19	D	38374	146224	895,180,535	263,187,295	1,158,367,830	88	1	6	8	2	17	Direct method mixed with stepdown method
20	E	114186	650301	5,944,017,002	1,937,397,089	7,881,414,091	291	1	17	22	25	65	Stepdown method

Besides the final department/function centers, there are indirect support cost centers on four levels. The level indicates at what stage the cost is allocated to other centers. Level 1 support cost mainly consists of one cost center, namely regional costs. This cost is always allocated first, also to other support cost centers, which indicates a step-down method [2]. Level 2 support cost centers vary from one to seventeen cost centers. Most units have IT-related costs in addition to those related to hospital administration. Apart from these two categories, there are further substantial variations. Some hospital units separate the quality department costs, financial department costs, HR costs, and/or energy. The organizational unit in Region E was found to have 17 Level 2 cost centers because their account sheet is on a regional level comprising several hospital units, the administrative costs of which are divided into separate categories, which resembles a more detailed micro-costing approach.

Generally, we identify that overhead costs, comprising regional and hospital administration, are melded with indirect costs that are typically activity-based support-center related. Thus, a clear distinction between overhead and indirect costs is not maintained. Level 1 costs are all allocated to the final cost centers using earnings before interest, taxes, depreciation, and amortization (EBITDA) as a cost driver. However, four hospitals allocate the full regional costs to Level 2 (with 100%), which are then distributed to the medical departments. Further, Level 2 costs typically use EBITDA as a cost driver. Level 1 costs comprise a total of 55% of the support center costs and 13% of the total costs. Therefore, the coding and allocation method has a significant influence on the department costs. For some departments, a standardized cost allocation may alter their expenses substantially.

Level 3 cost centers comprise indirect costs. Hence, these support functions are more easily defined by activity and, thereby, differently distributed to the medical departments depending on the activity (i.e., service usage). The cost drivers applied are typically bed days, which are mainly used for allocating kitchen costs; time usage and square meters are also frequently applied, with the latter factor being used to allocate technical department costs. Level 4 cost centers mainly apply actual activity as an indicator for distributing costs. Examples of Level 4 cost centers are blood banks, immunization, patient hotels, clinical genetic departments, pharmacies, and anesthesia (Table 2).

**Table 2:** cost mapping

**COST MAPPING**	Service/administrative costs	L1: Regional admin costs L2: Common costs Administration Centre administration L3: Replacement corps Technical department Kitchen Cleaning Laundry L4: Clinical support dept. e.g. Immunological dept. Patient Hotel
	Final Cost Centre: clinical departments or functions	Hospital departments often divided into - Outpatient - Admissions (Beds) - Intensive care - Surgery

Although the national guideline instructs the hospital units to apply a step-down model in allocating costs, five out of twenty organizational units apply a significant amount of the Level 1 and 2 costs directly to the departments. Thus, the cost allocation method is not aligned across hospital units, and neither are cost center definitions.

The direct cost methodology cannot be explicitly identified in the cost accounts because this would demand service identification, which is separate information in the patient administrative and clinical databases. This analysis shows that the cost estimation lacks detail because cost is not registered per patient or diagnosis. Hence, there is a lack of activity-based costing registration. Thereby, and according to Tan and co-workers’ [12] conceptual framework, we can identify the accuracy of valuation of hospital services as being on a highly aggregate level. Assuming a particularly accurate service identification level, a top-down micro-costing method is applied. Thus, averages are applied in valuing patient services. This approach contradicts the findings of Chapman et al. [13], who state that Denmark applies a bottom-up patient-level costing approach; this is not the case when evaluating the cost allocation foundation. The cost accounts are linked to patients at a later stage, combining these analyzed cost data with patient activity data. Thus, the approach is performed at an aggregate level in the central organization of the Health Data Authority.

### Detailed department examples from the cost accounts

To describe the content of the cost accounts more effectively, we will illustrate one surgical function from two different cost account sheets. Table 3 depicts an overview of this function where we illustrate a university hospital function and a collective rural regional hospital’s cost account on the same function. The total costs of cost accounts show that all of the organizations have almost a similar level of costs. However, we see substantial differences in the eye surgery function total costs where the university hospital accounts for a much higher level of costs than the rural hospitals. From the national patient registry, we have retrieved total surgeries per function in order to compare this variable to the total costs. Here, we witness that the university hospital only has approximately 25% more surgeries than the combined region E hospitals. These numbers, both costs and surgeries, are excluding outpatient surgeries and costs. Although, we also see that the costs of the university hospital are 300% higher than region E. We find one explanation for this skewness in the cost account set-up where region E has separated eye implant surgeries from the other surgeries. Yet, adding region E implant surgery costs of DKK 3,542,488 still keeps region E costs dramatically lower than the university hospital. These costs are matched with patient activity when calculating the DRG rates, where we find 29 DRG rates within the inpatient eye surgery area. Calculating DRG averages from such extreme cost level differences clearly distorts the basis (the production value incorporated in the budget) on which the departments are held accountable.

**Table 3:** overview of one department/functions from two different cost accounts

Data source		University Hospital example	Regional Hospitals example
Cost accounts	Total costs	DKK 6,659,327,305	DKK 7,881,414,091
Cost accounts	Function detailed entries (items) of direct costs	139	108
	Total hospital entries (items)	11,360	16,876
**Eye surgery – department example**^a^			
Cost accounts	Direct costs (see Additional file 2 for details)	DKK 45,275,942 (79.16%)	DKK 11,679,801 (75.77%)^b^
Cost accounts	Indirect costs (see Additional file 3 for details)	DKK 11,919,420 (20.84%)	DKK 3,735,016 (24.23%)
Cost accounts	Total costs for rate setting	DKK 57,195,362	DKK 15,414,817
National patient registry	Number of hospitalized surgeries^c^	1624	1287
National patient registry	Number of diagnosis (type of surgeries) reported	29	29

^a^ This is excluding out patient surgery

^b^ In this region, eye implant surgeries are separated out. These costs are only labelled as direct costs with the amount of DKK 3,542,488

^c^ Surgery categories with less than 5 patients are not included in these numbers due to the Danish Data Protection Act. Thus, the numbers would higher.

We further investigate the underlying entries for the total costs, and we find that eye surgery functions have 139 (university hospital) and 108 (regional hospitals) direct cost entries for 1 year illustrated, which accentuates a highly aggregated accounting foundation for determining diagnosis costs. Thus, there is no foundation for calculating objective diagnosis rates based on actual diagnosis costs. In the direct cost details (see Additional files 3 and 4), we show that salaries account for a large part of the direct costs: approximately 70% of the direct costs in region E and 50% of direct costs at the university hospital. Reviewing the direct costs, we also find a large entry of medicine costs (app. DKK 11 million) that account for 25% of the eye surgery function’s direct costs, which only account for 10% in region E. Therefore, these large cost differences indicate substantial variations in the type of surgeries performed. Yet, this is an intuitive assumption because the cost accounts simply do not provide us with details to make such conclusions.

Reviewing the indirect costs (see Additional files 1 and 2), we also witness large discrepancies where region E has substantially more detailed data than the university hospital. Region E has 12 indirect cost centers allocating costs to the surgery function, whereas the university hospital only has 8 indirect cost centers. Additionally, region E allocate the indirect costs according to well-defined cost drivers, such as square meters, purchases, and gross margin. These cost drivers are evaluated as mostly well defined because they illustrate a reasonably logical link between the cost and the consumption of the cost, which is a prerequisite for correct service cost allocations according to accounting theory [1, 2]. The university hospital does not apply cost drivers but labels the costs as direct, which an administrative hospital and regional costs cannot be. Thus, the allocation applied by the university hospital indicates a large arbitrary space. Finally, the costs’ accounts and the distribution of costs show a lack of capacity description and relation. Rather, we observe a full cost model where all costs are allocated without knowing the capacity, particularly employee time. This issue prevents appropriate planning both locally and centrally in the distribution of funds, treatment requirements, and treatment activity. It is particularly this challenge that TDABC overcomes, which is one of the reasons for recommending its application in hospital cost analysis and planning.

### Possibility of TDABC

**Fig. 2:**
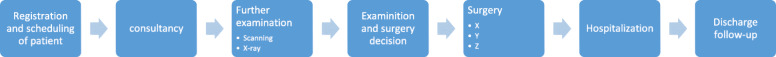
fictive care delivery value chain

We now take departure from the available data and, from that, seek to understand how more beneficial and transparent data could provide the basis for correct decision-making. According to the seven steps provided in the methods section, we first need to select the (step 1) medical condition. In the above eye surgery example, we know that there are 29 surgical processes (diagnosis) just within that function. Thus, we now examine the processes rather than the diagnosis as such. Several elements of the processes for the 29 types of eye surgeries would possibly be the same, and therefore this method could easily be applied, although a given department handles several types of surgeries and treatments. Secondly, we need to (step 2) define the care delivery value chain. A fictional simple eye surgery value chain is provided in Fig. 2.

Each process can contain detailed cost information, but the TDABC exercise seeks to simplify this information. For example, “hospitalization” may include laundry of bed sheets, attending nurse, equipment, and other costs. The idea is that the next step (step 3) should provide a more detailed process map that shows all the different variations of possible activities. Kaplan [6] provides a process map that further takes into account the possibilities of, for example, 60% of the patients will require an X-ray after the first consultation. This type of process map would increase the complexity of the value chain, but it will add insights into all types of activities performed. For example, the initial consultation process could include the physician, filling in documents, clinical assistant, ambulant service, equipment applied (equipment room – if, for example, scanning or X-ray applies). The surgical procedure would become more detailed and describe physician time, anesthesiologist, nurse(s), and operating theater. All cost drivers are time. Then, hospitalization would map the registered nurse’s x time per day, bed x days, food (kitchen) x meals/days, etc.

In our eye surgery data above, we identified large differences in the costs registered. For example, the university hospital had salary costs of DKK 21,600,116 (adding the salary multiplied by allocated rate from Additional file 3), whereas region E salary costs amounted to DKK 7,031,918 (adding the salary multiplied by allocated rate from Additional file 4). Although the difference in aggregated number of surgeries is 25%, the salary level differences are 200% larger at the university hospital than region E. This difference calls for a recommended bottom-up approach to determine the processes per hospital department. When the processes have been locally developed, they can be centrally compared. The possibility of comparing data further calls for separating part of the indirect costs, such as regional and hospital administration, to avoid skewing the data. Some of the indirect data, however, such as laundry, cleaning, etc. (mainly levels 3 and 4 data from the current cost accounts) can be applied as a direct cost and incorporated into the TDABC cost rates.

Thus, the central issue in developing a process map displaying the activities is (step 4) defining the time that each activity takes and (step 5) estimating the cost of each resource, which is better done locally. In the above-depicted eye surgery functions, we found that salaries accounted for up to 70% of all direct costs. This cost can easily be calculated for the time applied if more details are provided. This detail should involve the number of physicians, nurses, other staff, and their specific salaries. One important part of these calculations is the calculation of capacity. This is a central part of TDABC, which differentiates this method from the original ABC [2, 61].

This is the tool that can provide department and hospital managers with insights into how to plan capacity. In Table 4, we show a fictional example of a physician’s hours and costs as well as an X-ray room. We deduct 20% for meetings and other activities as a general approximation for staff [61]. We do the same for the X-ray room for cleaning and maintenance. Then, it is straightforward to estimate the capacity of each resource and calculate the capacity rate, as shown in Table 5. The next step is to decide on how much time is provided for each activity. For example, a consultation with a physician takes 15 min in general and, thereby, costs 15 × 12.08 = DKK 181.2. An X-ray normally takes 10 min and, therefore, costs 10 × 0.3 = 3.00 DKK (here, we need to add the radiologist’s salary). Finally, when these estimates have been provided, a (step 7) patient cost flow can be deducted.

**Table 4:** example of capacity and capacity cost rate calculation

**Physician hours**			**X-ray room and equipment**			
7.5 h per day	7.50		15 h per day	15.00		
5 days per week	5.00		7 days per week	7.00		
46 weeks per year	46.00		52 weeks per year	52.00		
**Total hours per year**	**1725.00**	theoretical capacity	**Total hours per year**	**5460.00**	theoretical capacity	
Practical capacity (80%)	1380.00	hours	Practical capacity (80%)	4368.00	hours	
Annual cost of a physician	1,000,000.00	DKK	Depreciation of equipment	300,000.00	DKK	
			Building costs /rent	200,000.00	DKK	
Cost per physician hour	724.64	DKK	Utilities	5000.00	DKK	
Cost per physician minute	12.08	DKK	Cleaning	30,000.00	DKK	
			Administration (scheduling)	10,000.00	DKK	
			**Total cost per X-ray room per year**	**545,000.00**	**DKK**	
			Cost per X-ray room hour	18.17	DKK	
			Cost per X-ray room minute	0.30	DKK

**Table 5:** Accounting technical differences

	Current cost account system (DRG foundation)	Recommended cost account system (TDABC)
Cost drivers	Gross margins, square meters, number of purchases etc., or negotiated rates	Nursing time, physician time, operating theater time, in-patient days, clinical care units (representing time based measures of the acuity of treatment offered to different types of patients)
Activity cost pools	Support departments, clinical departments	Performed activities: consultation, filling documents, registering patient data, hospitalization, hotel services, care (nursing care given)
Cost data	Aggregated cost data	Detailed cost data on function level (e.g. salary on professional level)
Calculations	Wide national averages: Sum of total costs within a function (including overhead) divided with more detailed activity according to points Resource cost (C) = Ʃ cost / number of activities	Detailed local resource calculations: Simple formula: Resource cost (C) = Quantity of resource units (Q) x Price per unit of resource (P) More specifically becomes: Actual Costs for patient “i” in episode period “j”: Cij = c1j*X1ij + c2j*X2ij + c3j*X3ij + … + cNj*XNij Where: • Xnij = the number of units of resource n consumed by patient i in episode period j. • Cni = the unit cost of resource n episode period j.

Taking a starting point from the Danish hospitals current cost account system, the cost for each department and/or function needs to be calculated for each activity where staff time and room/equipment time would be the starting point. Having defined this capacity enables us to apply the costs and calculate the cost rates per activity. However, to do so demands changes to the set-up of cost accounting in order for it to be feasible. In the following section, we will depict the main differences to understand how this can possibly be handled.

### DRG national averages versus TDABC

In Table 5, we display the technical differences between the current cost account system and the demand for a TDABC. Not surprisingly, the TDABC demands more local detailed accounting data, which warrants an increase in the micro-costing bottom-up approach versus the current gross-costing top-down approach [12] in guiding the cost data filling.

Taking a bottom-up approach will further increase capacity insights and, thus, autonomy and controllability [6, 7, 9]. Ultimately, the focus on a more detailed application of cost accounting data will improve the accuracy of these data and, therefore, decision-making, which furthermore increases the ability to prioritize and become more cost efficient [5, 13]. Kaplan [6] highlights the clinical input as being essential where TDABC is performed by teams of clinicians, administrators, and finance staff, therefore making this process highly actionable. On the contrary, typical hospital cost allocation systems, such as DRG costing, are led and updated by finance departments where clinicians do not understand how costs are assigned.

The Danish regions are currently investigating the possibilities of incorporating value-based healthcare, including cost accounting [45, 47, 62]. Expert groups have been appointed and reports written [46, 62]. They have concluded that the current top-down approach and the existing cost account database are feasible for a budget setting and national comparative analysis and for calculating possible efficiency initiatives. Our analysis shows that this is not the case. Overall, three areas of cost account challenges can be identified in our assessment: 1) the hospital structure in Denmark influencing the cost account reporting and, thereby, 2) the usage of different cost center definitions as well as allocation methods, and finally, 3) the inclusion of overhead costs and indirect costs with direct costs and the implications of aggregated costing data. Thus, basing budgets and resource allocation on production values derived from the current cost database distorts hospital capabilities and decision-making because it does not provide transparent insights or information regarding their contributions; rather, their services are melded with 19 other approaches and treatment mixes.

## Discussion

In recent years, we have witnessed an increasing interest in a value-based agenda [25, 26, 35–37, 63, 64] based on the ideas of Porter, Kaplan, and others [21, 22, 29] with a developing focus on the costing techniques of TDABC [4, 9, 16, 27, 28]. This trend is also seen in the Danish healthcare system [25, 47, 55], and as stated by Triantafillou (2020), referring to the national patient registry, “Only very few countries in the world have a population whose dealing with the public sector is so intensely and systematically monitored and registered as in Denmark” ( [25] , p. 6), which has been supported by other studies [54]. However, there is a collective agreement on the lack of cost accounting information for the purpose of supporting a value-based healthcare agenda [25, 46]. Thus, an investigation of the current cost accounts, their implications, and their future applicability is relevant to understand the required changes.

Steinmann et al. [26] highlight that the concept of VBHC is ambiguous, particularly the idea of patient value, which is perceived as the patient–doctor relationship experience in the Netherlands rather than a strategic goal for healthcare as the theory proposes, which has connotations with Groenewoud and colleagues’ [65] concerns about the ethical drawbacks of VBHC where they highlight a neglecting of patients’ intrinsic values. Triantafillou [57] has similar concerns in a Danish setting as is further highlighted by other studies. Although we do not investigate the strategic part of VBHC, we pursue the cost accounting, which despite the strategic focus, is essential in steering and prioritizing activities in health because cost accounting lays the foundation for essential decision-making [2, 5]. In fact, the transparent application of cost accounting procedures may support a better focus on valuable activities rather than distorted and skewed data, which increase pressure on healthcare staff [66]. Thus, we believe that this type of accounting technique assessment is valuable despite the strategic focus. In fact we believe that better and more transparent cost data will provide better ethical choices and possibilities, because it will support clinically valuable decision-making due to its focus on controllability and capacity steering. Controllability is defined as the degree of influence a manager has on costs, revenue, and other items the manager is accountable for [1, 2]. It is part of responsibility accounting where the idea is knowledge and transparency rather than control as such [67]. Yet, our assessment is limited to the cost accounting prepared and send to the national Health Data Authority. Thus, we do not have insights into local hospital accounting registration systems, i.e. the underlying entries of for example the aggregate items of staff costs. However, we know that the underlying cost registry systems are decoupled from the patient registry systems and that the match of this data is performed at the national Health Data Authority level [43] as illustrated in Fig. 1. Therefore, we are able to perform an assessment of the cost data provided for national healthcare decision-making which is following cascaded down to the regions and hospitals through production-value requirements and budgets.

Our findings show that the current cost accounting system is skewed and distorts influence, knowledge, and thus decision-making, both centrally and locally. The application of wide averages for DRG rates further applied for reimbursement purposes or benchmarking has long been criticized for its promotion of competitive and unethical contemplation [66, 68] due to the ability to maximize resource allocation and establishing a gaming element within healthcare [66, 68–70] but also due to its distorted underlying data consisting of wide averages [12, 71]. In our particular assessment, such skewness is exemplified by Region E, which only has one set of cost accounting reports compared to our example of a university hospital. We identified large differences in their cost account allocations and level of information as well as cost levels altogether. Within one region, one specialization may be mixed with other specializations in the cost accounting set-up, whereas these specializations may be separated in another region. For example, we witnessed how the eye surgery in Region E was divided into two functions, whereas it was all melded into one function at the university hospital. Thus, due to hospital heterogeneity, a skewness in the allocation of costs exists, supported by the fact that the cost weights do not reflect the hospital treatment. This issue is illuminated by Ankjær-Jensen et al. [31], who claim major uncertainty in the statement of cost centers at the individual hospitals. This present evaluation identified continued uncertainty 10 years later. We further note a structural variation, which may substantiate this uncertainty. Different organizational set-ups challenge standardization. Thus, a contradiction appears in the central wish from the Health Ministry and the Health Data Authority to align cost accounting aiming at accomplishing cost-effective and transparent decision-making in resource allocation, which consequently holds hospitals accountable for specific activity levels distorted by organizational and regional structural differences. This practice contradicts the controllability principle, and thus would ultimately demotivate managers due to lack of insights and ability to provide information based decision-making [67].

Finally, melding overhead and indirect costs with direct costs influences cost transparency. This fact further alters the ability for department management to influence measures upon which the departments are accounted for. Although these practices are common in DRG cost accounting, the practices contradict some of the basic management accounting principles that are essential in the value-based agenda [4]. It is only direct and indirect costs (i.e., levels 3 and 4 support costs in the empirics) that vary with actual patient activity in the medical departments. Levels 1 and 2 costs are administrative overhead in nature, and they are decoupled from department activity. When these types of costs are allocated to departments, it implies a full costing system [43] and not an activity-based system, as illustrated by Kaplan and Witkowski [4]. Thereby, the medical departments lack influence on the patient activity costing. Additionally, activity-based costing requires that direct and indirect costs are separated according to the cost objects’ use of resources [4, 72]. In the current cost accounts from the Danish hospitals, this is not the case. All costs are allocated on a department level, therefore implying a highly aggregated cost information system with no primary relationship between the patient service and actual costs. The cost accounts, therefore, allocate service activity on an organizational rather than detailed level, which is required for activity-based costing [72].

Thus, there is a lack of standardization in the cost accounting foundation, both for the DRG rate calculations that influence fairness in the following application of DRGs for benchmarking but particularly for future patient-level cost initiatives. Therefore, we suggest TDABC as a solution to more transparent and bottom-up led cost registration, which according to the literature, has proven to have ample opportunities in healthcare [7, 16, 27, 28, 38, 40]. Kaplan et al. [8] conducted four case studies on TDABC and found that one of the major barriers for implementing TDABC, in fact, was the misaligned fee-for-service reimbursement system, which encouraged high-cost and potential inefficient care, which has connotations to the Danish current cost account foundation for the calculation of production values. Thus, although TDABC is highlighted to be a bottom-up driven solution [4], also by the Danish Regions [62], central changes to cost calculation, cost registration, and thus the cost account database is vital to avoid distortion and demotivation of initiating local initiatives. Rather, an opportunity exists for central healthcare decision-makers to enable cost registry simultaneously and at the same detailed level as the national patient registry known for its detailed and insightful non-financial activity data [54].

## Conclusions

We studied all cost accounts from Danish hospitals in 2015. These cost accounts lay the foundation for different types of calculations, such as DRG rates. The accounting numbers are implemented in calculations used for benchmarking, resource allocation, and management control. Therefore, these numbers are significant and have substantial implications for decision-making. Our objective was to examine these available hospital cost accounting information to understand current hospital- and sector-level implications as well as the feasibility for future value-based healthcare application. We find that the exceedingly aggregate hospital department-level cost data are not tied to patient nor diagnostic information, hence contradicting policy intentions both regarding DRGs as well as value-based healthcare. We find large structural variations in the different hospital cost accounts, distorting their applicability for national standard measures as well as local transparent decision-making. Finally, overhead and indirect costs are melded with direct costs, distorting department managers’ accountable ability. Therefore, to improve insightful decision-making, we propose and encourage a substantially more practical emphasis on costing systems at the hospital level with the possible implementation of TDABC. Hospital accounting reports and other calculative reports on the hospital level are often assumed to be correct [66, 71]; however, we identify remarkable discrepancies that ultimately influence decision-making. For hospitals to become better informed and more cost efficient, a significantly more detailed cost account system is essential. This examination is, therefore, not only directed to the research society on healthcare costing but should also act as informational for policy-makers and hospital managers, an approach that is further called for by Chapman [14].

A limitation to our study is the lack of experimentation with TDABC locally to showcase this method in a practical manner. However, we have focused on explaining the drawbacks of the current cost account system to highlight the need for future changes. Subsequent research could, in fact, investigate more specific applications of TDABC and its potential influence on decision-making. Such studies may also analyze department-level costing data in more detail or qualitatively pursue an understanding of the underlying incentive system. Additionally, it is necessary to develop better holistic accounting solutions for public healthcare systems and inform decision-makers about DRG’s limitations and the detailed costing requirements for implementing patient-level cost data.

## Supplementary Information

**Additional file 1.**

**Additional file 2.**

**Additional file 3.** University hospital direct cost overview for surgical function.

**Additional file 4.** Region direct cost overview for surgical function.

## Data Availability

The datasets used and analyzed during the current study are available from the corresponding author on reasonable request.

## Abbreviations

TDABC: Time-driven activity based costing
DRG: Diagnosis related group
VBHC: Value based healthcare

## Footnotes

1 These yearly reports can be found on the Danish Health Ministry’s webpage in their files of publications searching for “productivity” https://sum.dk/publikationer?query=produktivitet assessed on May 31st 2021.
