# Supplementary material for: From centralized DRG costing to decentralized TDABC-assessing the feasibility of hospital cost accounting for decision-making in Denmark
Source: BMC Health Serv Res. 2021 Aug 18;21:835. doi: 10.1186/s12913-021-06807-4 (PMC8371815; doi:10.1186/s12913-021-06807-4)
Supplement: Supplementary file 3 — Additional file 3. University hospital direct cost overview for surgical function. [file 12913_2021_6807_MOESM3_ESM.docx]

Additional file 3: university hospital direct cost overview for surgical function

|  |  |  |  | Final |
| --- | --- | --- | --- | --- |
| **Account #** | **text** | **account name** |  | Eye surgery |
|  |  | **TOTAL DIRECT COST** |  | **45,275,942** |
|  |  |  | Cost absolute numbers | percentage allocated |
| 3017121404 | MEDICIN | TRANSFERRED PURCHASES | 30 | 6 |
| 3349119004 | CONSULTANTS (ASSISTING PATIENTS AND CITIZENS) | SERVICES WITHOUT TAX | 3,550 | 100 |
| 3349141050 | OTHER PHYSICIAN ITEMS | OTHER PURCHASES | 311,745 | 100 |
| 3349321601 | MEDICOTECHNICAL EQUIPMENT | ENTREPRENEUR- AND WORKMAN SERVICES | 32,310 | 100 |
| 3332141006 | OTHER PHYSICIAN ITEMS | OTHER PURCHASES | 193,523 | 100 |
| 3347112150 | OTHER PURCHASES | OTHER PURCHASES | 244 | 100 |
| 3347141002 | OTHER PHYSICIAN ITEMS | OTHER PURCHASES | 6,326 | 100 |
| 3347321604 | MEDICOTECHNICAL EQUIPMENT | ENTREPRENEUR- AND WORKMAN SERVICES | 920 | 100 |
| 3348111050 | OFFICE EXPENSES | OTHER PURCHASES | 406 | 100 |
| 3348112103 | OTHER PURCHASES | OTHER PURCHASES | 4,036 | 100 |
| 3348112405 | OTHER PURCHASES | TRANSFERRED PURCHASES | 1,368 | 100 |
| 3348119000 | CONSULTANTS (ASSISTING PATIENTS AND CITIZENS) | SERVICES WITHOUT TAX | 4,970 | 100 |
| 3348141006 | OTHER PHYSICIAN ITEMS | OTHER PURCHASES | 5,760 | 100 |
| 3348315209 | OTHER ACQUISITIONS | OTHER PURCHASES | 211 | 100 |
| 3348321608 | MEDICOTECHNICAL EQUIPMENT | ENTREPRENEUR- AND WORKMAN SERVICES | 12,656 | 100 |
| 3334111007 | OFFICE EXPENSES | OTHER PURCHASES | 1,680 | 100 |
| 3334141003 | OTHER PHYSICIAN ITEMS | OTHER PURCHASES | 182,351 | 100 |
| 3334152102 | THE STATES SERUMINSTITUTE | SERVICES WITHOUT TAX | 13,643 | 100 |
| 3334321605 | MEDICOTECHNICAL EQUIPMENT | ENTREPRENEUR- AND WORKMAN SERVICES | 64,464 | 100 |
| 3334322709 | FURNITURE AND EQUIPMENT | OTHER SERVICES | 1,537 | 100 |
| 3314131001 | IMPLANTS | OTHER PURCHASES | 12,986 | 100 |
| 3314141007 | OTHER PHYSICIAN ITEMS | OTHER PURCHASES | 1,583,742 | 100 |
| 3314311204 | MEDICOTECHNICAL EQUIPMENT | OTHER PURCHASES | 3,586 | 100 |
| 3314315250 | OTHER ACQUISITIONS | OTHER PURCHASES | 1,743,700 | 100 |
| 3314321609 | MEDICOTECHNICAL EQUIPMENT | ENTREPRENEUR- AND WORKMAN SERVICES | 27,386 | 100 |
| 3430141006 | OTHER PHYSICIAN ITEMS | OTHER PURCHASES | 218,033 | 100 |
| 3430321608 | MEDICOTECHNICAL EQUIPMENT | ENTREPRENEUR- AND WORKMAN SERVICES | 234,022 | 100 |
| 3430325506 | OTHER OPERATION AND MAINTENANCE | OTHER PURCHASES | 1,456 | 100 |
| 3324119204 | CONSULTANTS (ASSISTING PATIENTS AND CITIZENS) | SALG AF PRODUKTER AND YDELSER | -9,652 | 100 |
| 3324141005 | OTHER PHYSICIAN ITEMS | OTHER PURCHASES | 543,737 | 100 |
| 3324141102 | OTHER PHYSICIAN ITEMS | SERVICES WITHOUT TAX | -7,654 | 100 |
| 3324321607 | MEDICOTECHNICAL EQUIPMENT | ENTREPRENEUR- AND WORKMAN SERVICES | 218,589 | 100 |
| 3433102303 | MEETINGS | OTHER SERVICES | 14,620 | 100 |
| 3433792601 | OTHER | OTHER INDTÆGTER | -61,332 | 100 |
| 3346141009 | OTHER PHYSICIAN ITEMS | OTHER PURCHASES | 43,675 | 100 |
| 3326021201 | SEMINARS | OTHER SERVICES | 3,730 | 100 |
| 3326111006 | OFFICE EXPENSES | OTHER PURCHASES | 117 | 100 |
| 3326141002 | OTHER PHYSICIAN ITEMS | OTHER PURCHASES | 365,805 | 100 |
| 3326141150 | OTHER PHYSICIAN ITEMS | SERVICES WITHOUT TAX | 25,883 | 100 |
| 3326152101 | THE STATES SERUMINSTITUTE | SERVICES WITHOUT TAX | 1,231 | 100 |
| 3326311250 | MEDICOTECHNICAL EQUIPMENT | OTHER PURCHASES | 13,074 | 100 |
| 3326321604 | MEDICOTECHNICAL EQUIPMENT | ENTREPRENEUR- AND WORKMAN SERVICES | 11,252 | 100 |
| 3326325707 | OTHER OPERATION AND MAINTENANCE | ENTREPRENEUR- AND WORKMAN SERVICES | 5,500 | 100 |
| 3426141050 | OTHER PHYSICIAN ITEMS | OTHER PURCHASES | 153,425 | 100 |
| 3426311207 | MEDICOTECHNICAL EQUIPMENT | OTHER PURCHASES | 8,551 | 100 |
| 3426321601 | MEDICOTECHNICAL EQUIPMENT | ENTREPRENEUR- AND WORKMAN SERVICES | 35,464 | 100 |
| 3443152104 | THE STATES SERUMINSTITUTE | SERVICES WITHOUT TAX | 21,868 | 100 |
| 3333141050 | OTHER PHYSICIAN ITEMS | OTHER PURCHASES | 3,156,498 | 100 |
| 3333311207 | MEDICOTECHNICAL EQUIPMENT | OTHER PURCHASES | 22,256 | 100 |
| 3333321601 | MEDICOTECHNICAL EQUIPMENT | ENTREPRENEUR- AND WORKMAN SERVICES | 30,444 | 100 |
| 3442141001 | OTHER PHYSICIAN ITEMS | OTHER PURCHASES | 21,588 | 100 |
| 3296011206 | SALARY | SALARY |  | 38.9826199 |
| 3296011206 | SALARY | SALARY |  | 38.9826199 |
| 3296011206 | SALARY | SALARY |  | 38.9826199 |
| 3296011206 | SALARY | SALARY | 34,891,923 | 38.9826199 |
| 3296011303 | SALARY | MANUEL ITEMS FROM ACCOUNTING SYSTEM | 124,527 | 38.9826199 |
| 3296011400 | SALARY | TRANSFERRED SALARYNINGER | 600,000 | 38.98 |
| 3296012350 | TEMP. AGENCIES | TRANSFERRED SERVICES | 1,175 | 38.98 |
| 3296013004 | BUSINESSTRAVEL/ TRANSPORT | SERVICES WITHOUT TAX | 58,160 | 38.9826199 |
| 3296013101 | BUSINESSTRAVEL/ TRANSPORT | OTHER SERVICES | 746 | 38.9826199 |
| 3296014000 | BUSINESSTRAVEL | SERVICES WITHOUT TAX | 5,568 | 38.98 |
| 3296014108 | BUSINESSTRAVEL | OTHER SERVICES | 423 | 38.98 |
| 3296015007 | OTHER PERSONNEL COST | OTHER PURCHASES | 161,458 | 38.98261991 |
| 3296015104 | OTHER PERSONNEL COST | SERVICES WITHOUT TAX | 46,255 | 38.98261991 |
| 3296015201 | OTHER PERSONNEL COST | OTHER SERVICES | 3,996 | 38.98261991 |
| 3296015406 | OTHER PERSONNEL COST | SALG AF PRODUKTER AND YDELSER | -720,816 | 38.98261991 |
| 3296015600 | OTHER PERSONNEL COST | FOOD | 300 | 38.98261991 |
| 3296021007 | SEMINARS | OTHER PURCHASES | 6,980 | 38.98261991 |
| 3296021104 | SEMINARS | SERVICES WITHOUT TAX | 157,504 | 38.98261991 |
| 3296021201 | SEMINARS | OTHER SERVICES | 257,702 | 38.98261991 |
| 3296021805 | SEMINARS | TRANSFERRED SERVICES | 9,530 | 38.98261991 |
| 3296022208 | FURTHER TRAINING, PHYSICIANS | OTHER SERVICES | 1,432 | 38.98 |
| 3296100101 | PHONE, POSTAGE, INTERNET | OTHER SERVICES | 16,393 | 38.98261991 |
| 3296100403 | PHONE, POSTAGE, INTERNET | TRANSFERRED SERVICES | 39,459 | 38.98261991 |
| 3296102007 | MEETINGS | FOOD | -6,952 | 38.98261991 |
| 3296102104 | MEETINGS | OTHER PURCHASES | 388 | 38.98261991 |
| 3296102309 | MEETINGS | OTHER SERVICES | -1,813 | 38.98 |
| 3296102503 | MEETINGS | TRANSFERRED PURCHASES | 50,945 | 38.98261991 |
| 3296103003 | OTHER SERVICES | SERVICES WITHOUT TAX | 1,335 | 38.98261991 |
| 3296103100 | OTHER SERVICES | OTHER SERVICES | 252 | 38.98 |
| 3296111006 | OFFICE EXPENSES | OTHER PURCHASES | 126,576 | 38.98261991 |
| 3296111103 | OFFICE EXPENSES | SERVICES WITHOUT TAX | 541 | 38.98 |
| 3296112002 | OTHER PURCHASES | FOOD | 22,608 | 38.98261991 |
| 3296112150 | OTHER PURCHASES | OTHER PURCHASES | 92,804 | 38.98261991 |
| 3296112401 | OTHER PURCHASES | TRANSFERRED PURCHASES | 52,080 | 38.98261991 |
| 3296113009 | REPRESENTATION | OTHER PURCHASES | 1,063 | 38.98 |
| 3296114005 | PATIENTTRANSPORT | SERVICES WITHOUT TAX | 4,122 |  |
| 3296116601 | LAUNDRY | TRANSFERRED SERVICES | 174,646 | 38.98261991 |
| 3296119007 | CONSULTANTS (ASSISTING FOR PATIENTS AND CITIZENS) | SERVICES WITHOUT TAX | 17,571 | 35.93 |
| 3296119104 | CONSULTANTS (ASSISTING FOR PATIENTS AND CITIZENS) | OTHER SERVICES | 49,627 | 20.74 |
| 3296119201 | CONSULTANTS (ASSISTING FOR PATIENTS AND CITIZENS) | SALG AF PRODUKTER AND YDELSER | -12,820 | 38.98 |
| 3296121400 | MEDICIN | TRANSFERRED PURCHASES | 32,757,445 | 38.98261991 |
| 3296141002 | OTHER PHYSICIAN ITEMS | OTHER PURCHASES | 1,385,122 | 38.98 |
| 3296141150 | OTHER PHYSICIAN ITEMS | SERVICES WITHOUT TAX | 48,897 | 38.98261991 |
| 3296151008 | CLINICAL ANALYSIS | SERVICES WITHOUT TAX | 72,050 | 38.98261991 |
| 3296151350 | CLINICAL ANALYSIS | OTHER SERVICES | 163 | 38.98261991 |
| 3296151709 | CLINICAL ANALYSIS | TRANSFERRED SERVICES | 393,083 | 38.98261991 |
| 3296151806 | CLINICAL ANALYSIS | INTERNE INDTÆGTER | 2,450 | 38.98 |
| 3296152101 | THE STATES SERUMINSTITUTE | SERVICES WITHOUT TAX | 226,792 | 38.98261991 |
| 3296311102 | MEDICOTECHNICAL EQUIPMENT | ACQUISITIONS | 0 | 38.98 |
| 3296311250 | MEDICOTECHNICAL EQUIPMENT | OTHER PURCHASES | 74,482 | 38.98261991 |
| 3296312206 | FURNITURE AND EQUIPMENT | OTHER PURCHASES | 35,794 | 38.98261991 |
| 3296314209 | IT EQUIPMENT | OTHER PURCHASES | 115,226 | 38.98261991 |
| 3296315205 | OTHER ACQUISITIONS | OTHER PURCHASES | 40,256 | 38.98261991 |
| 3296321604 | MEDICOTECHNICAL EQUIPMENT | ENTREPRENEUR- AND WORKMAN SERVICES | 208,217 | 38.98261991 |
| 3296322600 | FURNITURE AND EQUIPMENT | ENTREPRENEUR- AND WORKMAN SERVICES | 3,216 | 38.98 |
| 3296322708 | FURNITURE AND EQUIPMENT | OTHER SERVICES | 3,105 | 38.98261991 |
| 3296323402 | KØRETØJER | FUEL AND PROPELLANT | 7,163 | 31.72 |
| 3296323607 | KØRETØJER | SERVICES WITHOUT TAX | 362 | 27.84 |
| 3296323704 | KØRETØJER | ENTREPRENEUR- AND WORKMAN SERVICES | 8,714 | 19.08 |
| 3296323801 | KØRETØJER | OTHER SERVICES | 29,006 | 0.214 |
| 3296324409 | IT-EQUIPMENT | OTHER PURCHASES | 18,796 | 38.98 |
| 3296324603 | IT-EQUIPMENT | ENTREPRENEUR- AND WORKMAN SERVICES | 3,882 | 38.98261991 |
| 3296324700 | IT-EQUIPMENT | OTHER SERVICES | 52,800 | 38.98261991 |
| 3296325502 | OTHER OPERATION AND MAINTENANCE | OTHER PURCHASES | 4,712 | 38.98 |
| 3296325707 | OTHER OPERATION AND MAINTENANCE | ENTREPRENEUR- AND WORKMAN SERVICES | 12,826 | 38.98261991 |
| 3296325804 | OTHER OPERATION AND MAINTENANCE | OTHER SERVICES | 3,925 | 38.98261991 |
| 3296406650 | INDVENDIG MAINTENANCE | TRANSFERRED SERVICES | 12,700 | 38.98 |
| 3296407002 | INSTALLATIONS, MAINTENANCE | OTHER PURCHASES | 4,408 | 38.98261991 |
| 3296407207 | INSTALLATIONS, MAINTENANCE | ENTREPRENEUR- AND WORKMAN SERVICES | 6,069 | 38.98 |
| 3296407401 | INSTALLATIONS, MAINTENANCE | TRANSFERRED SERVICES | 10,350 | 38.98 |
| 3296792607 | OTHER | OTHER INDTÆGTER | 0 | 38.98 |
| 3299011207 | SALARY | SALARY | 7,715,892 | 100 |
| 3299011207 | SALARY | SALARY |  | 100 |
| 3299013005 | BUSINESSTRAVEL/ TRANSPORT | SERVICES WITHOUT TAX | 1,615 | 100 |
| 3299013102 | BUSINESSTRAVEL/ TRANSPORT | OTHER SERVICES | 32 | 100 |
| 3299111007 | OFFICE EXPENSES | OTHER PURCHASES | 204 | 100 |
| 3299112100 | OTHER PURCHASES | OTHER PURCHASES | 762 | 100 |
| 3299112402 | OTHER PURCHASES | TRANSFERRED PURCHASES | 100 | 100 |
| 3299116602 | LAUNDRY | TRANSFERRED SERVICES | 1,783 | 100 |
| 3299141003 | OTHER PHYSICIAN ITEMS | OTHER PURCHASES | 387,473 | 100 |
| 3299315206 | OTHER ACQUISITIONS | OTHER PURCHASES | 6,000 | 100 |
| 3299321400 | MEDICOTECHNICAL EQUIPMENT | OTHER PURCHASES | 810 | 100 |
| 3299321605 | MEDICOTECHNICAL EQUIPMENT | ENTREPRENEUR- AND WORKMAN SERVICES | 27,990 | 100 |
| 3299324604 | IT-EQUIPMENT | ENTREPRENEUR- AND WORKMAN SERVICES | 2,850 | 100 |
| 3299407003 | INSTALLATIONS, MAINTENANCE | OTHER PURCHASES | 7,000 | 100 |
| YY8 |  |  | -150,000 | 38.98 |
| YY9 |  |  | -150,000 | 38.98 |
| YY10 |  |  | -2,550 | 38.98 |
