# Supplementary material for: From centralized DRG costing to decentralized TDABC-assessing the feasibility of hospital cost accounting for decision-making in Denmark
Source: BMC Health Serv Res. 2021 Aug 18;21:835. doi: 10.1186/s12913-021-06807-4 (PMC8371815; doi:10.1186/s12913-021-06807-4)
Supplement: Supplementary file 4 — Additional file 4. Region direct cost overview for surgical function. [file 12913_2021_6807_MOESM4_ESM.docx]

Additional file 4: Region direct cost overview for surgical function

| **Account #** | **text** | **account name** |  |  |
| --- | --- | --- | --- | --- |
|  |  |  |  | Final |
|  |  |  |  | Eye surgery |
|  |  |  |  | 2,213,216,005.0 |
|  |  |  |  | 11,679,801 |
|  |  |  | Cost absolute numbers | percentage allocated |
| 1255102202 | Personnel | Other purchases | 320.00 | 100 |
| 1255112909 | Personnel | Transferred services | 76,267.00 | 100 |
| 1255117900 | Personnel | Transferred services | 11,337.67 | 100 |
| 1255147907 | Medicin | Transferred purchases | 1,164,774.48 | 100 |
| 1255150908 | Medicin | Transferred services | 2,640.00 | 100 |
| 1255160202 | Other medical items | Other purchases | 6,250.00 | 100 |
| 1255161209 | Other medical items | Other purchases | 206.04 | 100 |
| 1255167207 | Other medical items | Other purchases | 4,265,989.81 | 52.94684 |
| 1255176400 | Medical analysis | Excl. tax | 3,068.61 | 100 |
| 1255299200 | Material- and activity expenses | Other purchases | 6,684.85 | 100 |
| 1255609250 | Acquisitions, equipment | Acquisitions (more than DKK100.000) | - | 100 |
| 1255610208 | Acquisitions, equipment | Other purchases | 252,500.00 | 100 |
| 1255640204 | Operations and maintenance | Other purchases | 11,101.40 | 100 |
| 1255651400 | Operations and maintenance | Entrepreneur- and workman services | 3,021.05 | 100 |
| 1255657409 | Operations and maintenance | Entrepreneur- and workman services | 205,032.50 | 100 |
| 1255706450 | Operations and maintenance | Entrepreneur- and workman services | 10,832.26 | 100 |
| 1255720208 | Material- and activitiesexpenses | Other purchases | 12,978.47 | 100 |
| 1255722200 | Material- and activity expenses | Other purchases | 4,602.00 | 100 |
| 1255723207 | Material- and activity expenses | Other purchases | 207,198.22 | 100 |
| 1255772208 | Material- and activity expenses | Other purchases | 884.32 | 100 |
| 1255904902 | Material- and activity expenses | Other services | 550.00 | 100 |
| 1255920207 | Material- and activity expenses | Other purchases | 2,292.56 | 100 |
| 1255922250 | Material- and activity expenses | Other purchases | 9,469.63 | 100 |
| 1256069403 | Seminars | Excl. tax | 1,025.00 | 100 |
| 1256070401 | Seminars | Other services | 46.00 | 100 |
| 1256150901 | Medicin | Transferred services | 666.00 | 100 |
| 1257010100 | Salary |  | 674,977.33 | 17.06121166 |
| 1257020106 | Salary |  | (9,058.62) | 17.06121166 |
| 1257030101 | Salary |  | 1,086,000.25 | 17.06121166 |
| 1257032104 | Salary |  | (127,904.33) | 17.06121166 |
| 1257036150 | Salary |  | (9,876.00) | 17.06121166 |
| 1257060450 | Seminars | Excl. tax | - | 17.06121166 |
| 1257060809 | Payments - other regions | Payment to regions | 550.85 | 17.06121166 |
| 1257061406 | Seminars | Other services | 7,000.00 | 17.06121166 |
| 1257069407 | Seminars | Excl. tax | 14,684.80 | 17.06121166 |
| 1257077450 | Seminars | Excl. tax | - | 17.06121166 |
| 1257084406 | Personnel | Excl. tax | 1,257.61 | 17.06121166 |
| 1257086409 | Personnel | Excl. tax | 1,819.40 | 17.06121166 |
| 1257096404 | Personnel | Excl. tax | 12,764.43 | 17.06121166 |
| 1257098407 | Personnel | Excl. tax | 1,071.99 | 17.06121166 |
| 1257123800 | Personnel | Transferred services | 3,000.00 | 17.06121166 |
| 1257844400 | Material- and activitiesexpenses | Food | 1,836.00 | 17.06121166 |
| 1257880903 | Material- and activitiesexpenses | Transferred purchases | 12,566.00 | 17.06121166 |
| 1258030105 | Salary |  | 6,920,874.84 | 24 |
| 1258032108 | Salary |  | (202,290.47) | 24 |
| 1258036103 | Salary |  | (100,694.38) | 24 |
| 1258055450 | Salary | Other services | 750,769.76 | 24 |
| 1258060403 | Seminars | Excl. tax | 7,064.00 | 24 |
| 1258061450 | Seminars | Other services | 2,750.00 | 24 |
| 1258061906 | Seminars | Transferred services | 6,700.00 | 24 |
| 1258086402 | Personnel | Excl. tax | 13,663.58 | 24 |
| 1258095800 | Material- and activitiesexpenses | Other purchases | 4,588.00 | 24 |
| 1258102203 | Personnel | Other purchases | 799.20 | 24 |
| 1258117901 | Personnel | Transferred services | 2,056.83 | 24 |
| 1258167208 | Other medical items | Other purchases | 139.00 | 24 |
| 1258643409 | Operations and maintenance | Entrepreneur- and workman services | 191.50 | 24 |
| 1258651401 | Operations and maintenance | Entrepreneur- and workman services | 1,981.40 | 24 |
| 1258671208 | Acquisitions, equipment | Other purchases | 1,902.00 | 24 |
| 1258673200 | Acquisitions, equipment | Other purchases | 5,615.03 | 24 |
| 1258674304 | Acquisitions, equipment | Other purchases | 14,217.00 | 24 |
| 1258706400 | Operations and maintenance | Entrepreneur- and workman services | 15,033.90 | 24 |
| 1258772209 | Material- and activitiesexpenses | Other purchases | 56,196.83 | 24 |
| 1258774201 | Material- and activitiesexpenses | Other purchases | 280.00 | 24 |
| 1258904903 | Material- and activitiesexpenses | Other services | 16,852.48 | 24 |
| 1258922200 | Material- and activitiesexpenses | Other purchases | 11,823.66 | 24 |
| 1259010108 | Salary |  | 22,246,906.00 | 24 |
| 1259012100 | Salary |  | (315,478.93) | 24 |
| 1259016106 | Salary |  | (320,206.98) | 24 |
| 1259017900 | Salary | transferred salary | (275,796.86) | 24 |
| 1259018907 | Salary | transferred salary | (278,765.76) | 24 |
| 1259030109 | Salary |  | 477,645.86 | 24 |
| 1259060407 | Seminars | Excl. tax | 2,632.94 | 24 |
| 1259061403 | Seminars | Other services | 492.31 | 24 |
| 1259061950 | Seminars | Transferred services | 3,950.00 | 24 |
| 1259066405 | Seminars | Excl. tax | 83,808.62 | 24 |
| 1259067401 | Seminars | Other services | 358.80 | 24 |
| 1259069404 | Seminars | Excl. tax | 90,485.97 | 24 |
| 1259070402 | Seminars | Other services | 11,464.30 | 24 |
| 1259072405 | Seminars | Excl. tax | 19,337.30 | 24 |
| 1259073401 | Seminars | Other services | 15,520.00 | 24 |
| 1259077407 | Seminars | Excl. tax | 6,800.12 | 24 |
| 1259081404 | Seminars | Other services | 1,190.40 | 24 |
| 1259086406 | Personnel | Excl. tax | 5,988.94 | 24 |
| 1259098404 | Personnel | Excl. tax | 1,608.00 | 24 |
| 1259102207 | Personnel | Other purchases | 646.56 | 24 |
| 1259117905 | Personnel | Transferred services | 3,486.58 | 24 |
| 1259140508 | Personnel | Other income | - | 24 |
| 1259161203 | Other medical items | Other purchases | 2,492.30 | 24 |
| 1259167201 | Other medical items | Other purchases | 245,181.14 | 24 |
| 1259299205 | Material- and activitiesexpenses | Other purchases | 10,771.01 | 24 |
| 1259317203 | Material- and activitiesexpenses | Other purchases | 1,943.90 | 24 |
| 1259544404 | Operations and maintenance | Other services | 8,539.90 | 24 |
| 1259607201 | Acquisitions, equipment | Other purchases | 303.20 | 24 |
| 1259610202 | Acquisitions, equipment | Other purchases | 80,727.30 | 24 |
| 1259640209 | Operations and maintenance | Other purchases | 13,864.99 | 24 |
| 1259643402 | Operations and maintenance | Entrepreneur- and workman services | 3,325.00 | 24 |
| 1259651405 | Operations and maintenance | Entrepreneur- and workman services | 10,738.00 | 24 |
| 1259657403 | Operations and maintenance | Entrepreneur- and workman services | 29,283.00 | 24 |
| 1259674308 | Acquisitions, equipment | Other purchases | 2,145.00 | 24 |
| 1259720202 | Material- and activity expenses | Other purchases | 11,521.50 | 24 |
| 1259723201 | Material- and activity expenses | Other purchases | 2,644.59 | 24 |
| 1259772202 | Material- and activity expenses | Other purchases | 7,014.31 | 24 |
| 1259774205 | Material- and activity expenses | Other purchases | 160.00 | 24 |
| 1259777409 | Material- and activity expenses | Excl. tax | 1,480.00 | 24 |
| 1259830202 | Material- and activity expenses | Food | 4,186.68 | 24 |
| 1259904907 | Material- and activity expenses | Other services | 2,029.51 | 24 |
| 1259920201 | Material- and activity expenses | Other purchases | 8,418.26 | 24 |
| 1259922204 | Material- and activity expenses | Other purchases | 250.01 | 24 |
